# Ghrelin Stimulation of Growth Hormone-Releasing Hormone Neurons Is Direct in the Arcuate Nucleus

**DOI:** 10.1371/journal.pone.0009159

**Published:** 2010-02-11

**Authors:** Guillaume Osterstock, Pauline Escobar, Violeta Mitutsova, Laurie-Anne Gouty-Colomer, Pierre Fontanaud, François Molino, Jean-Alain Fehrentz, Danielle Carmignac, Jean Martinez, Nathalie C. Guerineau, Iain C. A. F. Robinson, Patrice Mollard, Pierre-François Méry

**Affiliations:** 1 Inserm U-661, Montpellier, France; 2 CNRS UMR 5203, Institut de Génomique Fonctionnelle, Montpellier, France; 3 Université Montpellier 1, 2, Montpellier, France; 4 Division of Molecular Neuroendocrinology, MRC National Institute for Medical Research, The Ridgeway, Mill Hill, London, United Kingdom; 5 CNRS UMR 5247, Institut des Biomolécules Max Mousseron, Montpellier, France; University of Texas Health Science Center, United States of America

## Abstract

**Background:**

Ghrelin targets the arcuate nucleus, from where growth hormone releasing hormone (GHRH) neurones trigger GH secretion. This hypothalamic nucleus also contains neuropeptide Y (NPY) neurons which play a master role in the effect of ghrelin on feeding. Interestingly, connections between NPY and GHRH neurons have been reported, leading to the hypothesis that the GH axis and the feeding circuits might be co-regulated by ghrelin.

**Principal Findings:**

Here, we show that ghrelin stimulates the firing rate of identified GHRH neurons, in transgenic GHRH-GFP mice. This stimulation is prevented by growth hormone secretagogue receptor-1 antagonism as well as by U-73122, a phospholipase C inhibitor and by calcium channels blockers. The effect of ghrelin does not require synaptic transmission, as it is not antagonized by γ-aminobutyric acid, glutamate and NPY receptor antagonists. In addition, this hypothalamic effect of ghrelin is independent of somatostatin, the inhibitor of the GH axis, since it is also found in somatostatin knockout mice. Indeed, ghrelin does not modify synaptic currents of GHRH neurons. However, ghrelin exerts a strong and direct depolarizing effect on GHRH neurons, which supports their increased firing rate.

**Conclusion:**

Thus, GHRH neurons are a specific target for ghrelin within the brain, and not activated secondary to altered activity in feeding circuits. These results support the view that ghrelin related therapeutic approaches could be directed separately towards GH deficiency or feeding disorders.

## Introduction

The hypothalamic arcuate nucleus is a heterogeneous structure involved in the regulation of homeostasis. Its functions rely on the specific actions of its outputs; for example, growth hormone releasing hormone (GHRH) and somatostatin are involved in body growth [1], and neuropeptide Y (NPY) and agouti related peptide (AgRP) are involved in feeding [2]. The distribution of receptors and afferent nerve terminals within the arcuate nucleus are generally diffuse, supporting the view that afferent inputs coordinate combinations of outputs from this structure. Ghrelin, the endogenous growth hormone secretagogue [3], [4], is one such hypothalamic input. Indeed, ghrelin not only stimulates the growth hormone (GH) axis [1], but also induces feeding and modifies body energy consumption [5], [6], as well as modulating the gonadotropic axis [7]. The ghrelin receptor (GHSR, growth hormone secretagogue receptor-1) is found in several neuronal subtypes in the arcuate nucleus [8]–[11], where a diffuse pattern of ghrelin-containing terminals has been demonstrated [12].

Recent studies have addressed the organisation of this circuitry. In addition to its direct effects on the pituitary, ghrelin clearly targets GH release indirectly at the level of the arcuate nucleus since: 1) anatomical disconnections between the hypothalamus and the pituitary gland blunt GH secretion induced by GHS *in vivo* [13], [14]; 2) the GHSR is expressed in GHRH neurons, which trigger GH release by the pituitary gland [8]–[11]; 3) *in vivo* GHS treatments enhance GHRH secretion in sheep [1], [15] and induce *c-fos* expression in GHRH neurons in rodents [16]. Furthermore, ghrelin and GHS enhance the electrical activity of non-identified neurons in the arcuate nucleus [17]–[19], and ghrelin enhances calcium dynamics in isolated hypothalamic neurons, *in vitro* [20], [21]. While these results do not provide a specific mechanism of action, collectively they suggest that ghrelin exerts a direct effect at the level of GHRH neurons.

In contrast to this, other data suggest an indirect modulation of GHRH neurons by ghrelin. Indeed, the arcuate nucleus is intimately involved in the effects of ghrelin on the feeding circuits [5], [6], with NPY neurons appearing as central ghrelin sensors in this role [2], [22]. NPY neurons are the main ghrelin receptor (GHSR)-expressing cells of the arcuate nucleus [9], [10], and they upregulate *c-fos* expression in response to ghrelin perfusion [5]. NPY neurons signal through a complex release of NPY, AgRP, and γ-aminobutyric acid (GABA) [2], [22]. Accordingly, the orexigenic effect of ghrelin is absent in NPY/AgRP double knockout mice, despite unaltered growth and feeding [22], [23]. It is also attenuated in mice whose vesicular GABA transporter is specifically ablated in AgRP-expressing neurons [24]. *In vitro*, the stimulatory effect of ghrelin on NPY neurons orchestrates electrophysiological changes within the feeding circuits, including a GABAergic modulation of pro-opiomelanocortin (POMC) neurons and a dual GABA/NPYergic modulation of corticotrophin-releasing hormone (CRH) neurons [2], [12]. The role of NPY neurons may not be limited to the feeding circuits, per se, since GHRH neurons express NPY Y2 receptors which mediate the downregulation of GHRH mRNA induced by long term fasting in rodents [25], [26]. In addition, as NPY neurons often coexpress GABA [2], [12], [24], part of the GABAergic inputs to GHRH neurons [27] might originate from the NPY neurons themselves. Altogether, these findings suggest that NPY neurons might be the primary ghrelin sensors of the arcuate nucleus, funnelling information from within the feeding circuits to the GH axis.

Here, we took advantage of GHRH-GFP transgenic mice [28] to investigate whether ghrelin modulates GHRH neurons. We found that ghrelin stimulated the electrical activity of GHRH neurons in a direct manner, suggesting that parallel and apparently independent signalling at GHRH neurons and at NPY neurons can occur within the very restricted area of the arcuate nucleus. Our data support the view that ghrelin has multiple entries within the central nervous system. Thus, encoding of afferent information by the arcuate nucleus is not only supported by the identity of its outputs, the efferent neuropeptides, but also by the mechanism of action of its inputs, such as ghrelin, which can modulate the endocrine axis independently or in combination.

## Results

### Ghrelin Modulated the Firing Rate but Not the Firing Pattern of GHRH Neurons

**Figure 1:**
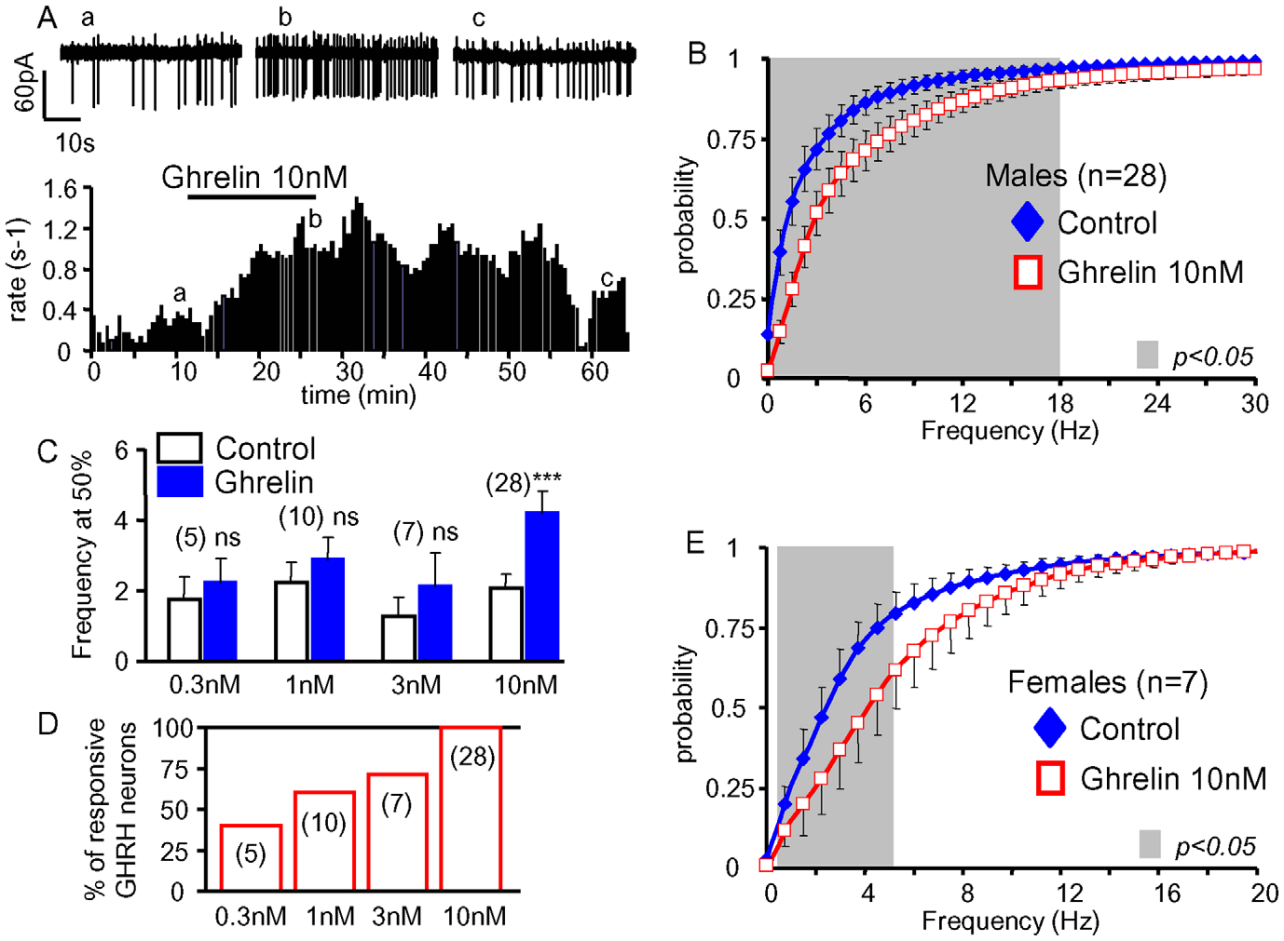
Ghrelin enhanced the activity of GHRH neurons. ***A***, time course of an experiment where the superfusion of a sagittal brain slice with 10 nM ghrelin increased, in a reversible manner, the rate of spontaneous action potentials of a GHRH neuron (individual traces shown on the top). ***B***, summary of the effects of ghrelin (10 nM) on the cumulative distributions of action potential frequencies in GHRH neurons from adult males; ***C***, mean effects of 0.3 to 10 nM ghrelin on the rate of spontaneous action potentials in GHRH neurons: the action potential frequencies observed at the half maximal values of the cumulated histograms were collected in each experiment in the absence and presence of ghrelin (see Methods for details). ***D***, the proportion of stimulatory effects induced by ghrelin increased in a dose-dependent manner in GHRH neurons. ***E***, summary of the effects of ghrelin (10 nM) on the distributions of action potential frequencies in GHRH neurons from adult females. In ***B & E***, the symbols and lines are the means and sem. Statistical differences (p<0.05, paired student-*t* test) between curves are framed by the grey areas. In ***D***, the bars and lines are the means and sem of the numbers of experiments indicated. ***, statistical difference from control values (p<0.001, paired student-*t* test).

We examined the effects of ghrelin on the electrical activity of identified GHRH neurons in brain slices from GHRH-GFP mice. In the experiment of Fig. 1A, spontaneous action potentials were first recorded under control conditions. Addition of 10 nM ghrelin to the external solution increased the firing rate from ∼0.2 to 0.9 Hz, and this stimulation disappeared during the washout of the peptide. The cumulative histograms of Fig. 1B summarize the results from similar experiments where the instantaneous frequencies of the spontaneous action potentials of GHRH neurons were compared under steady-state conditions in the absence and presence of 10 nM ghrelin (see Methods for additional information). The mean distribution under control conditions was shifted to the right (into the 0–18.5 Hz range) in the presence of ghrelin (grey area, n = 28, paired student's t-test, p<0.05). This increase in firing rate was also well described as an increase in the mean frequency at the half maximal values of the cumulated histograms (Fig. 1C). Lower concentrations of ghrelin (0.3–3 nM, n = 5 to 10) did not significantly change this parameter (Fig. 1C), and did not significantly shift the cumulative distribution of GHRH neuron action potentials (data not shown). However, 0.3–3 nM ghrelin occasionally enhanced the firing rate of GHRH neurons, and the proportion of responses increased in a concentration-dependent manner (Fig. 1D). Since 10 nM ghrelin always enhanced the electrical activity of GHRH neurons, the other effects of ghrelin were studied at this concentration.

The traces of Fig. 1A suggested that ghrelin did not change the firing pattern. Indeed, the mean skewness of the discharge density histograms was not changed by ghrelin (supporting Figure S1A). In accordance with the conclusion that ghrelin increases firing rates without changing the firing patterns of GHRH neurons, autocorrelogram analysis only showed differences in a very narrow range of action potential intervals (−0.3 to +0.3 s), (supporting Figure S1B–C). Because the GH axis exhibits several gender differences [1], the hypothalamic effect of ghrelin was then investigated in female mice. As summarized in Fig. 1E, ghrelin (10 nM) increased the electrical activity of all GHRH neurons tested from female GHRH-GFP mice (p<0.05 in the 0.75–6.25 Hz range, paired student's t-test), and did not change their firing pattern (data not shown). Thus, the stimulatory effect of ghrelin on GHRH neurons occurs in both sexes.

**Figure 2:**
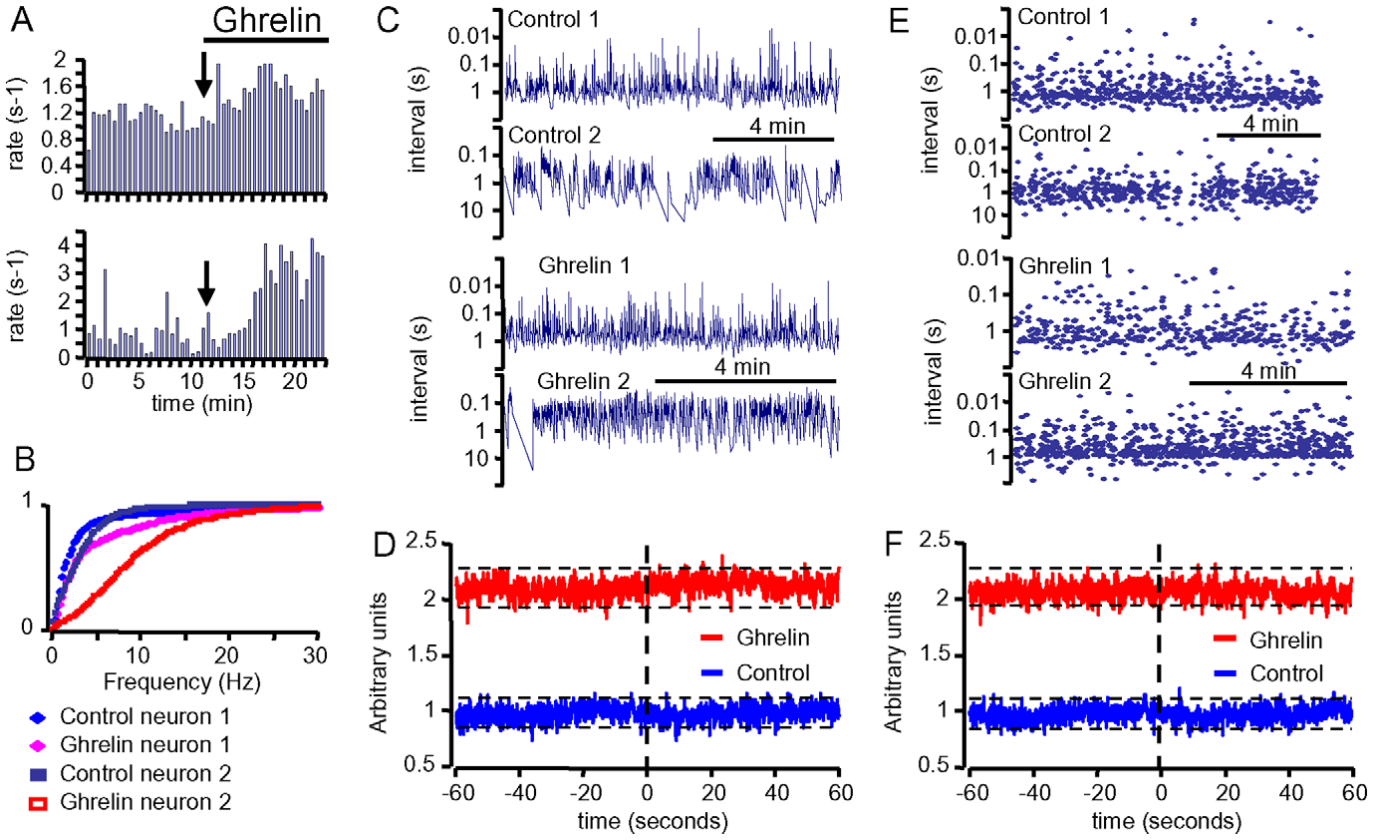
Ghrelin did not synchronize the activity of GHRH neurons in dual patch-clamp epxeriments. ***A***, stimulatory effects of ghrelin (10 nM) on the firing rate of two GHRH neurons recorded simultaneously. Action potential rates were calculated every 30 s. ***B***, cumulative distributions of the frequency of the action potentials of the GHRH neurons from panel ***A***, showing the extent of the rightward shifts induced by ghrelin. ***C***, intervals between action potentials of the GHRH neurons from panel ***A***, under control conditions and in the presence of ghrelin, were then used in generating the cross-correlograms shown in ***D***. The correlations of activity were calculated within consecutive bins of 100 ms during 60 s (see Methods for further details). Dotted lines indicate the 95% confidence boundaries within which the distributions behave as random, in the absence and presence of ghrelin. ***E&F***, same as ***C&D***, except that random distributions of instantaneous frequencies of action potentials were generated using the properties of the experimental data, in the absence and in the presence of ghrelin. The shapes of these cross-correlograms characterizing de-correlated series of events were almost undistinguishable from the experimental curves.

Because GHRH neurons are such a small population [2], [29], a GHRH releasing agent such as ghrelin (or ghrelin mimetics) might trigger synchronisation between GHRH neurons [15]. This synchronicity was then studied using the dual patch clamp technique. In the example of Fig. 2A, 10 nM ghrelin simultaneously enhanced the firing rates of two GHRH neurons. The cumulative distribution of the action potential frequencies of both neurons were shifted to the right by the peptide, though to different extents (Fig. 2B). This quantitative analysis was complemented with a qualitative analysis, where crosscorrelograms were computed (Fig. 2D), as described in the Methods section, using the stretches of spike trains recorded under steady-state conditions (Fig. 2C). In brief, the correlation between these spike trains consisted in counting the spikes of the neuron “2” at the specific time delay of 100 ms with respect to the spikes of the neuron “1”. The flat shape of the crosscorrelogram obtained under control conditions indicated that neuron “2” did not fire at a preferential time before/after neuron “1”. Thus, there was no correlation between the activities of the neurons. Ghrelin induced an upward shift in the distribution as expected for a stimulatory agent, but did not induce a distinctive peak in the cross-correlogram, suggesting independence between the activities of the two neurons. Both distributions were contained within the 95%-confidence boundaries of random distributions (dotted lines, computed as stated in Methods). Furthermore, random inter-event interval distributions (Fig. 2E) were generated using the distributions of the experimental sets of data (Fig. 2D), as described in Methods. They were used to model cross-correlograms between independent series of data (Fig. 2F), which were almost undistinguishable from the experimental results (Fig. 2D). These results were typical of six similar experiments, suggesting that ghrelin induced neither a hierarchy, nor a correlation of activity, amongst GHRH neurons.

### Pharmacological Profile of the Ghrelin Receptor

**Figure 3:**
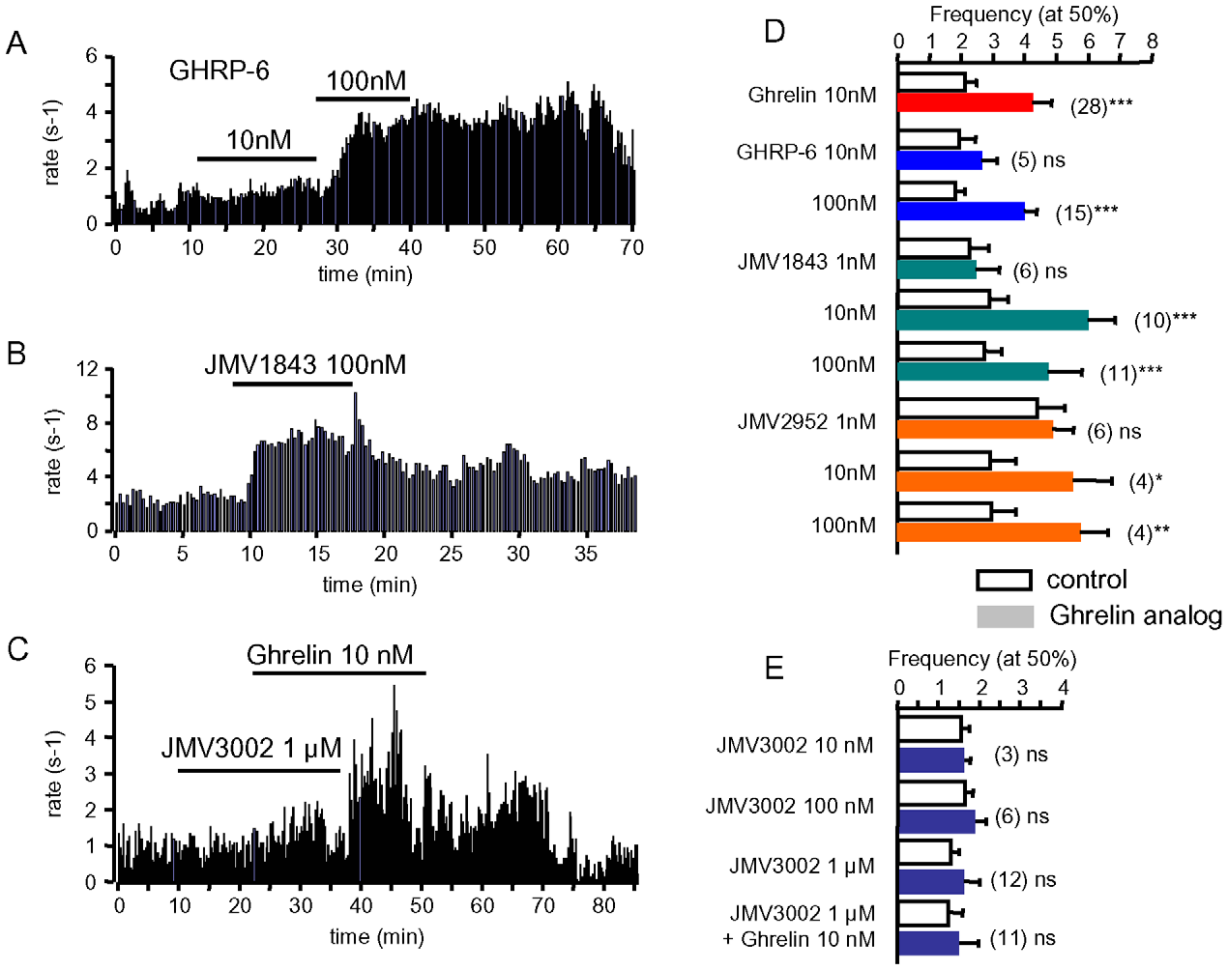
Effects of ghrelin receptor ligands on the activity of GHRH neurons. ***A to C***, typical experiments where superfusions with the agonists GHRP-6 (10 & 100 nM, ***A***) and JMV1843 (100 nM, ***B***) increased action potentials rates in adult male GHRH neurons; the antagonist JMV3002 (1 µM) prevented from the stimulatory effect of ghrelin (10 nM, ***C***). ***D–E***, summaries of the effects of GHSR agonists (***D:*** ghrelin, GHRP-6, JMV1843, JMV2952) or antagonist (***E:*** JMV3002) on the cumulative distributions of the spontaneous action potentials of GHRH neurons. The action potential frequencies observed at the half maximal values of the cumulated histograms were averaged according to the absence (white bars) and presence of agonists and/or antagonist (coloured bars, see Methods for details). In ***D&E***, bars and lines are the means and the sem of the numbers of experiments indicated. Statistical differences *, p<0.05; **, p<0.01; ***, p<.005 are shown (paired student-*t* test).

Prior to the discovery of ghrelin, it was established that GHS, such as GHRP-6, enhance the electrical activity of unidentified neurons in the arcuate nucleus [17], [18]. Like ghrelin, they exhibit a nanomolar affinity for GHSR, the canonical ghrelin receptor of the GH axis found in the arcuate nucleus [3], [4]. We therefore tested the effects of several GHS of differing structures. The electrical activity of a GHRH neuron (Fig. 3A, adult male) was enhanced by GHRP-6, slightly at 10 nM (from ∼0.9 to 1.4 Hz) and more strongly at 100 nM (to ∼3.7 Hz). This stimulatory effect on GHRH neurons was also observed when JMV1843 (10 nM), a potent *in vivo* GHSR agonist [30], [31], was superfused onto GHRH-GFP brain slices (Fig. 3B). Furthermore, while the GHSR antagonist, JMV3002 (1 µM) [33], did not change the activity of a GHRH neuron when applied alone (Fig. 3C), it blunted the effect of an addition of 10 nM ghrelin. The stimulatory effect of ghrelin developed upon washout of JMV 3002. The mean effects of the GHSs on the distribution of the frequencies of spontaneous action potentials of GHRH neurons were summarized in Fig. 3D and 3E. All the GHSR agonists, GHRP-6, JMV1843, and JMV2952 [32] increased the firing rate of GHRH neurons in a 1–100 nM range compatible with their affinities for GHSR (see mean frequencies at half maximal values of the cumulated histograms, Fig. 3D). JMV3002, the GHSR antagonist, was inactive on its own in the 10 nM to 1 µM range but significantly antagonized the stimulatory effect of 10 nM ghrelin (Fig. 3E). Hence, it is likely that GHSR activation mediates the enhancement of the electrical activity of GHRH neurons induced by ghrelin and the GHS tested in this study.

GHSR expression is seen early, at embryonic day 19 in the rat pituitary gland as well as in the brain [34], [35]. Accordingly, we found that ghrelin (10 nM) enhanced the firing rate of a GHRH neuron from immature, 6 day-old, male GHRH-GFP mice (supporting Figure S2A–B). Much later in life, aged individuals retain ghrelin-induced GH secretion as well as GHSR expression in the brain [4], [36]. The effect of ghrelin on GHRH neurons in aged (>22 months-old) male GHRH-GFP mice was indeed present but heterogeneous, being stimulatory in only 8 out of 13 experiments (supporting Figure S2C–D). Thus, the ghrelin responsiveness observed at different developmental stages in GHRH neurons, was compatible with the profile of GHSR expression in the brain [34]–[36].

### The Stimulation of GHRH Neurones by Ghrelin Requires Phospholipase C and Calcium Channels

**Figure 4:**
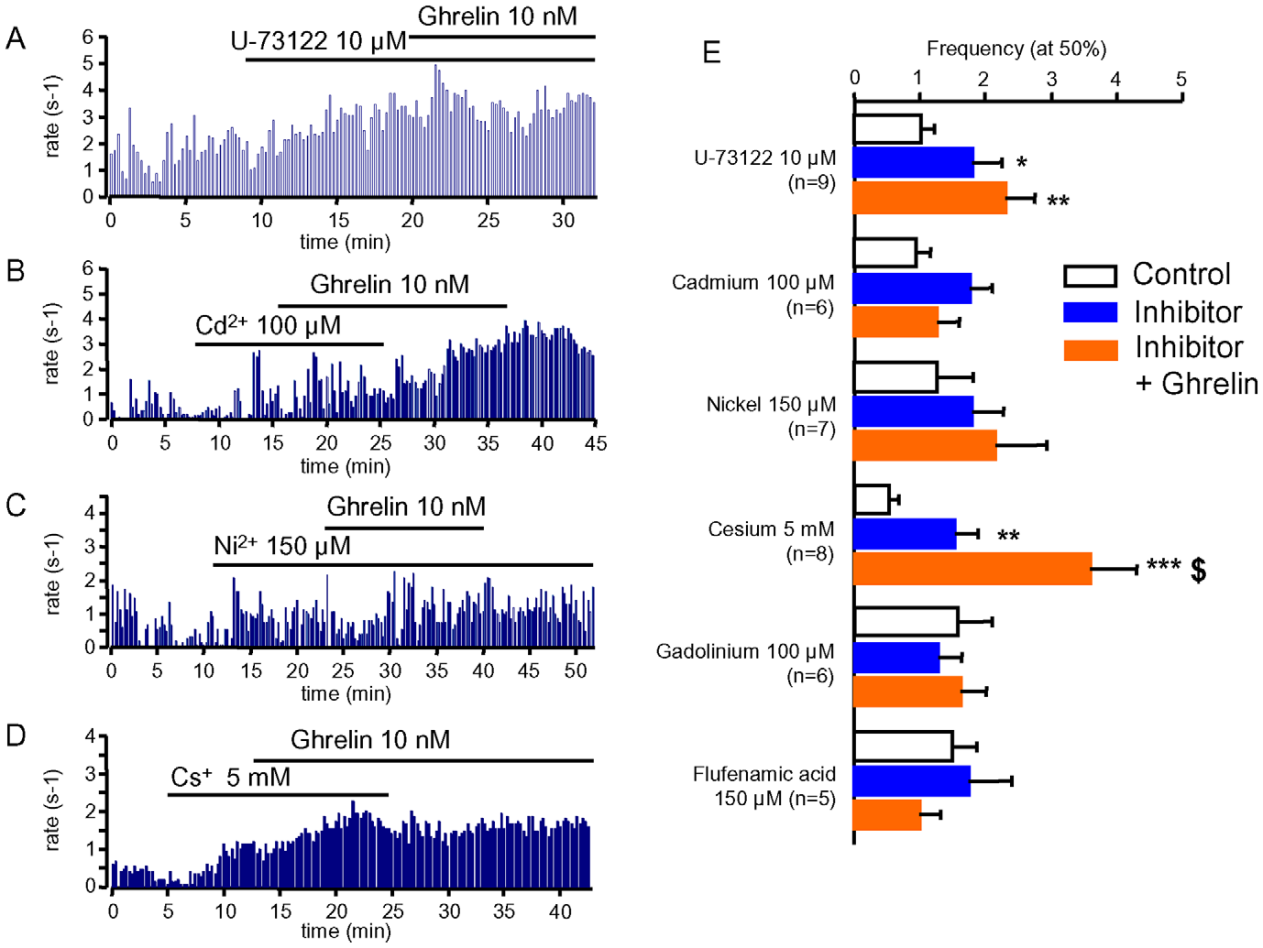
The effect of ghrelin on GHRH neurons requires phospholipase C and calcium channels. ***A–D***, typical recordings from GHRH neurons in the absence and presence of the phospholipase C inhibitor U-73122 (10 µM, ***A***); the high voltage-activated calcium channel blocker Cd^2+^ (100 µM, ***B***); the low voltage-activated calcium channel blocker Ni^2+^ (150 µM, ***C***); the *HCN* channel blocker Cs^+^ (5 mM, ***D***); and 10 nM ghrelin (***A–D***), as indicated by the lines. ***E***, summary of the effects of cellular signalling inhibitors on the cumulated distributions of spontaneous action potentials in GHRH neurons. The action potential frequencies observed at the half maximal values of the cumulated histograms were averaged according to the absence (white bars) and presence of inhibitor (blue bars), and in the presence of inhibitor plus 10 nM ghrelin (orange bars, see Methods for details). Bars and lines are the means and the sem of the numbers of experiments indicated. Statistical differences (*vs* control values *: p<0.05; **: p<0.01; ***, p<0.005; and *vs* inhibitor level $, p<0.05, paired student-*t* test) are shown.

The canonical effector of GHSR is phospholipase C dependent [4], [6], but GHSR activation by ghrelin can elicit the activation of other pathways depending on the tissue context [37]. The involvement of phospholipase C in GHRH neurons was examined first. Superfusion of a GHRH-GFP brain slice with 10 µM U-73122, a phospholipase C inhibitor [38]–[40], enhanced the firing rate of GHRH neurons, from ∼2 to 3.5 Hz, and this preincubation prevented the stimulatory effect of 10 nM ghrelin (Fig. 4A). In similar experiments, ∼10 minutes-long perfusion with U-73122 significantly increased the electrical activity of GHRH neurons and further addition of ghrelin had no significant effects in the presence of the phospholipase C inhibitor (Fig. 4E). In contrast, ghrelin enhanced the activity of GHRH neurons in the presence of U-73343 (10 µM, n = 4, data not shown), a U-73122 analog which does not inhibit phospholipase C activity [38]–[40].

Ion channels are the final effectors of ghrelin-stimulated pathways in various excitable cell types [4], [6], [20], [37], [41]. In addition, ghrelin tunes mitochondrial homeostasis and cellular energy supply in neurons [42]. Thus, the effect of ghrelin was first examined in the presence of a broad range inhibitor, namely flufenamic acid which inhibits several families of ionic channels and causes mitochondrial uncoupling [40], [43], [44]. As summarized in Fig. 4E, flufenamic acid (150 µM) fully antagonized the stimulatory effect of ghrelin in GHRH neurons. The role of ionic channels was further delimited. First, ghrelin did not enhance the electrical activity of GHRH neurons in the presence of Gd^3+^ (100 µM, Fig. 4E), a non-selective blocker of cationic channels including background channels or voltage dependent channels [41], [45]. In addition, Ni^2+^ (150 µM, Fig. 4B&E), a blocker of low voltage activated calcium channels [46], as well as Cd^2+^ (100 µM, Fig. 4C&E), a blocker of high voltage activated calcium channels [46], both prevented the stimulatory effects of ghrelin upon GHRH neurons. In contrast, extracellular Cs^+^ (5 mM, Fig. 4D&E) an inhibitor of the hyperpolarisation-activated cyclic nucleotides-gated cation channels (HCN) channels [44], significantly enhanced the electrical activity of GHRH neurons but did not antagonise the stimulatory effect of ghrelin. Therefore, ghrelin stimulates GHRH neurons in a phospholipase C and calcium dependent mechanism.

### The Effect of Ghrelin on GHRH Neurons Did Not Involve Somatostatin Input

**Figure 5:**
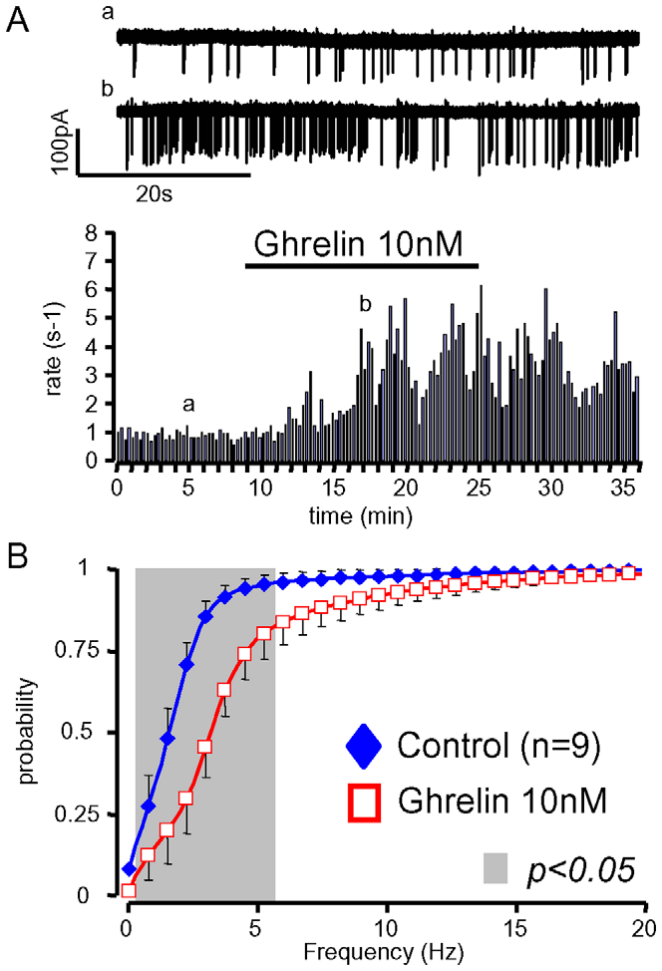
Ghrelin enhances the firing rates of GHRH neurons in the absence of somatostatin. ***A***, typical experiment where 10 nM ghrelin increased the firing rate of a GHRH neuron from an adult male somatostatin −/−, GHRH-GFP mouse (raw traces are shown on the top). ***B***, summary of the effects of ghrelin (10 nM) on the distributions of action potential frequencies in GHRH neurons from adult male somatostatin −/−, GHRH-GFP mice. Symbols and lines are the means and the sem of the numbers of experiments indicated. Statistical significances (p<0.05, paired student-*t* test, see methods) between curves are framed by the grey area.

Both GHRH neurons and somatostatinergic neurons express some GHSR [10]. The effect of ghrelin on the GH axis might require synaptic signalling between these two neuronal populations [1]. Accordingly, we took the opportunity to examine the effect of ghrelin on GHRH neurons in the absence of somatostatin, by breeding GHRH-GFP mice onto a somatostatin knockout mouse background [47] (a description of GHRH neurons of these animals is the subject of another submission). Fig. 5A shows that an identified GHRH neuron in an adult male somatostatin knockout mouse exhibited a spontaneous firing rate of ∼0.9 Hz under control conditions, increasing to ∼3.3 Hz upon addition of 10 nM ghrelin to the external solution. This stimulation was found in each experiment performed in GHRH neurons from GHRH-GFP X somatostatin null mice, and their mean spontaneous activity was significantly enhanced, as summarized in Fig. 5B (p<0.05 in the 0.5–6.5 Hz range, paired student's t-test where). A lower concentration of ghrelin (1 nM) had no significant effect (n = 3, data not shown). Thus, the activation of hypothalamic GHRH neurons by ghrelin occurs in the absence of somatostatin.

### The Effect of Ghrelin on GHRH Neurons Did Not Require NPY Neurotransmission

**Figure 6:**
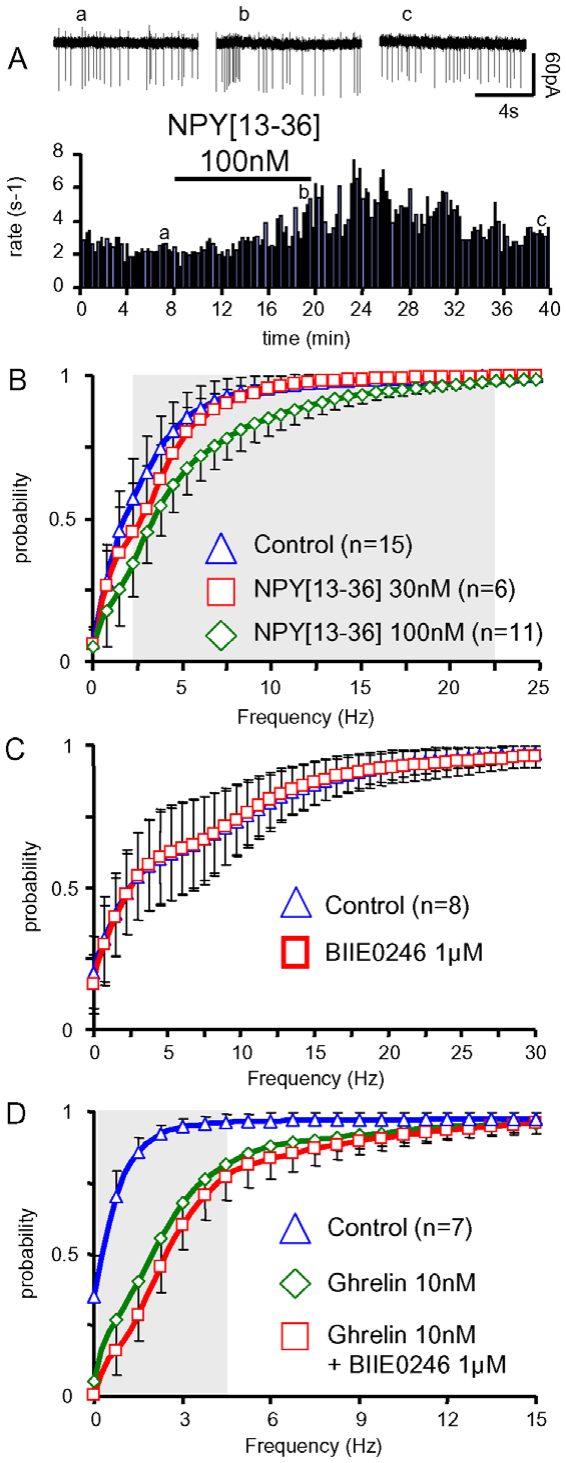
The effect of ghrelin on the firing rates of GHRH neurons did not require Y-2 receptors. ***A***, typical experiment where the Y-2 receptors agonist, NPY [13]–[36] 100 nM, increased, in a reversible manner, the spontaneous firing rate in a male GHRH neuron. Raw traces are shown on top of the panel. ***B***, summaries of the effects of NPY [13]–[36] (30 & 100 nM) on the distributions of action potential frequencies in GHRH neurons from adult male GHRH-GFP mice. ***C–D***, summaries of the effects of the Y-2 antagonist BIIE0246 alone (***C***) and of ghrelin in the absence or presence of BIIE0246 (***D***) on the distributions of action potential frequencies in GHRH neurons from adult male GHRH-GFP mice. Symbols and lines are the means and the sem of the numbers of experiments indicated. Statistical significance (p<0.05, paired student-*t* test) between curves (effect of ghrelin in the absence or presence of BIIE0246, ***D***) are framed by the grey area.

The NPY neurons are the predominant GHSR positive cells in the arcuate nucleus [9]–[11], and it is thought that NPY can modulate the GH axis, although the mechanisms are unclear [26]. We first tested a simple mechanism, whereby the NPY Y2 receptors, expressed by GHRH neurons [25], would mediate the effects of ghrelin. Interestingly, NPY [13]–[36] (100 nM), a selective NPY Y2 receptors agonist [48], increased the discharge rate of a GHRH neuron from an adult male (from ∼2.5 to 3.5 Hz, Fig. 6A). Like ghrelin, NPY [13]–[36] (100 nM) shifted the cumulated distribution of action potentials frequencies of GHRH neurons (p<0.05, in the 0.5–25 Hz range, Fig. 6B). The effect of a lower concentration (30 nM) of the Y2 receptor agonist was not significant.

The stimulatory effect of ghrelin was also examined in the presence of BIIE 0246, a selective NPY Y2 receptor antagonist [48]. On average, 1 µM BIIE 0246 did not change the activity of GHRH neurons in adult male mice (Fig. 6C), although it significantly blunted the stimulatory effect of 100 nM NPY [13]–[36] (n = 4, data not shown). Ghrelin induced significant rightward shifts of the distribution of the action potential frequencies (p<0.05, 0.25–4.75 Hz range for ghrelin + BIIE 0246, and 0.25–8 Hz for ghrelin, Fig. 6D), in the absence or presence of BIIE 0246 (p>0.05, ghrelin alone vs ghrelin + BIIE 0246). Therefore, Y2 receptor activation was not required for the stimulatory effect of ghrelin.

### The Stimulatory Effect of Ghrelin Did Not Require Fast Synaptic Transmission

**Figure 7:**
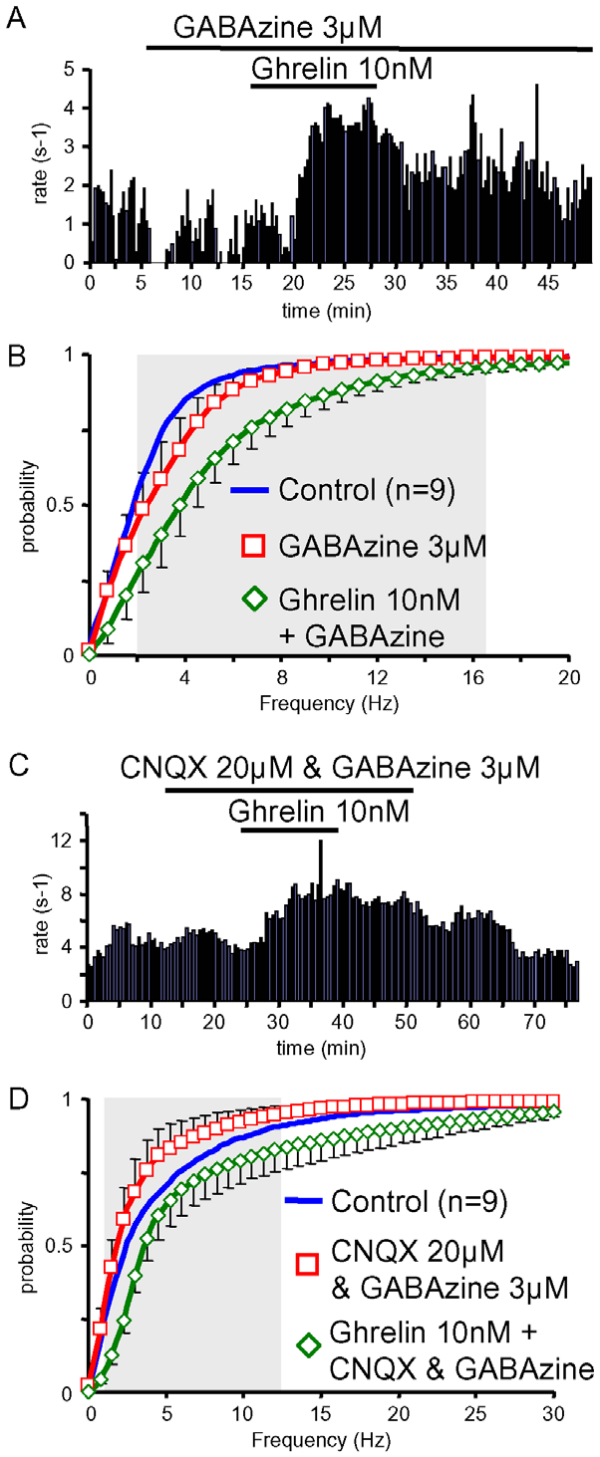
Ghrelin enhanced the firing rate of GHRH neurons during GABA_A_ receptor inhibition. ***A&B***, typical experiments where spontaneous action potentials of GHRH neurons were recorded as ghrelin 10 nM was applied in the continuing presence of 3 µM GABAzine, a GABA_A_ receptor antagonist, alone (***A***) or together with 20 µM CNQX, a AMPA/kainate receptors antagonist (***B***). ***C&D***, summaries of the stimulatory effect of ghrelin (10 nM) on the distributions of action potential frequencies in GHRH neurons in the presence of 3 µM GABAzine (***C***) or in the presence of 3 µM GABAzine + 20 µM CNQX (***D***). Symbols and lines are the means and the sem of the numbers of experiments indicated. Statistical significance (p<0.05, paired student-*t* test) between mean values recorded in the presence of inhibitors alone and in the presence of inhibitors plus ghrelin is framed by the grey area. Note that the mean control distributions are shown as lines and sem omitted, for clarity.

GABAergic neurotransmission by NPY neurons is intimately involved in the effects of ghrelin on CRH and POMC neurons [12]. GABA also modulates GHRH neurons [27], so its potential involvement in the effect of ghrelin on GHRH neurons was studied. Fig. 7A shows that 10 nM ghrelin strongly increased the firing rate of a GHRH neuron, in the continuing presence of an antagonist of ionotropic GABA_A_ receptors, GABAzine (4-[6-imino-3-(4-methoxyphenyl)pyridazin-1-yl] butanoic acid). On average, 3 µM GABAzine did not significantly modify the firing rates of GHRH neurons, because its effects were heterogeneous (Fig. 7B). Nevertheless, ghrelin shifted the distribution of action potentials frequencies in the presence of the GABA_A_ receptor antagonist (p<0.05, in the 2–17.5 Hz range). Thus, GABAergic neurotransmission was not necessary for the stimulatory effect of ghrelin on GHRH neurons.

Similarly, the involvement of glutamatergic neurotransmission in the effect of ghrelin was investigated, since this excitatory transmitter was strongly involved in the muscarinic modulation of GHRH neurons [27]. CNQX (6-cyano-7-nitroquinoxaline-2,3-dione), an antagonist at AMPA (α-amino-3-hydroxy-5-méthylisoazol-4-propionic acid) and kainate receptors was used in combination with GABAzine. In the experiment shown in Fig. 7C, the inhibitors slightly diminished the firing rate of the GHRH neuron (∼4.5 and 3.9 Hz in the absence and presence of CNQX + GABAzine, respectively), but the addition of ghrelin 10 nM, in the continuing presence of inhibitors, induced a robust increase in the firing rate of the neuron to ∼8.3 Hz. On average (Fig. 7D), the combination of GABAzine + CNQX tended to weaken the activity of GHRH neurons, though in a non significant manner, and ghrelin shifted the distribution of action potentials frequencies in the presence of the inhibitors (p<0.05, 1–12.5 Hz range). Thus the stimulatory effect of ghrelin did not require AMPA/kainate neurotransmissions.

### Ghrelin Did Not Modify Synaptic Currents in GHRH-GFP Neurons

**Figure 8:**
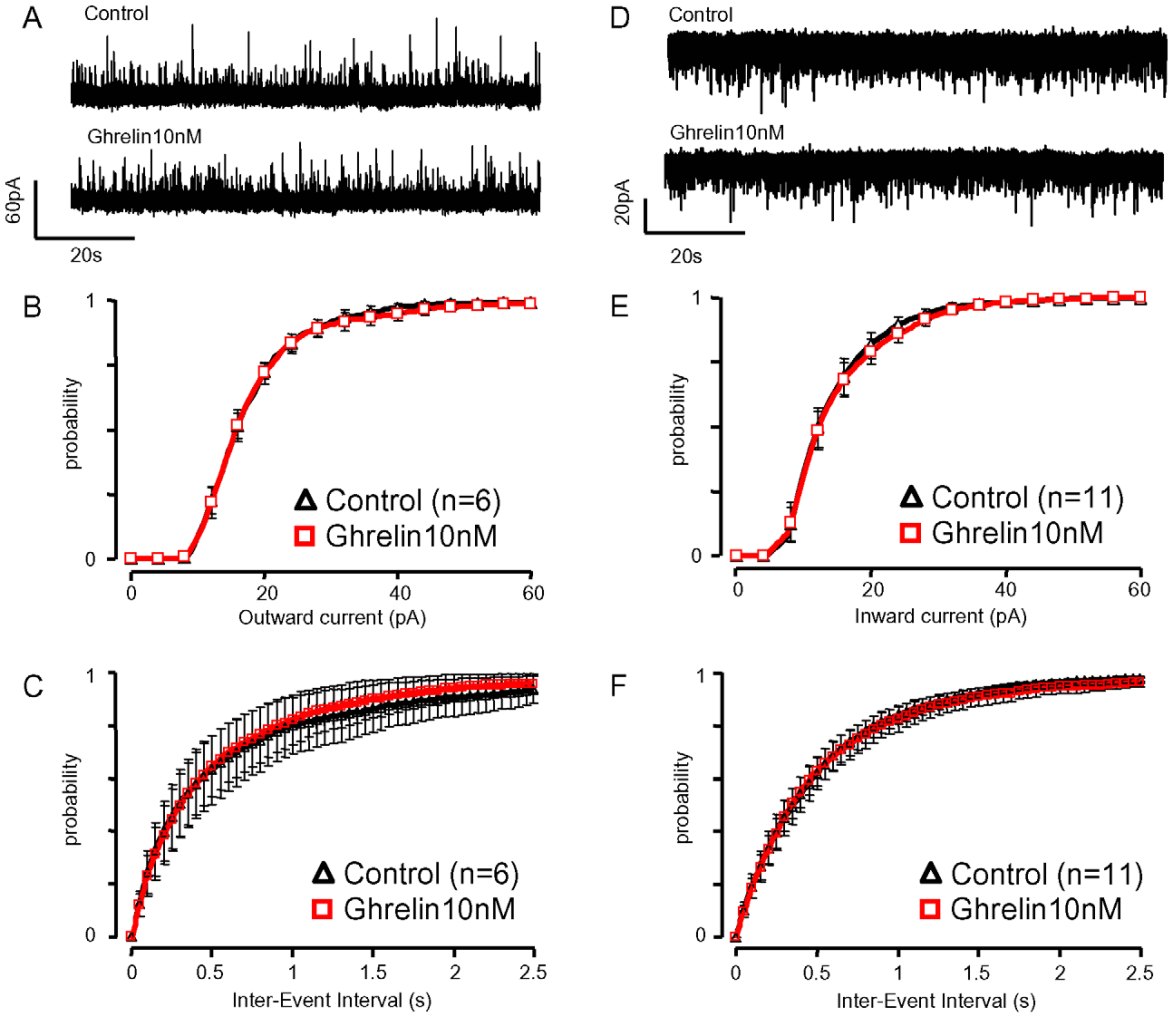
Ghrelin did not modify spontaneous synaptic currents of GHRH neurons. ***A&D***, raw traces of spontaneous glutamatergic (−30 mV) and GABAergic (−70 mV) synaptic currents recorded in the absence and presence of 10 nM ghrelin in GHRH neurons from adult male GHRH-GFP mice. The effects of ghrelin (10 nM) were summarized, on the amplitude (***B&E***) and intervals (***C&F***) of glutamatergic (***B&C***) and GABAergic (***E&F***) synaptic currents. The cumulative distributions are represented as symbols and lines, i.e. the means and the sem of the numbers of experiments indicated.

A modulation of GHRH neuron synaptic currents might play a subtle role in the effect of ghrelin. The spontaneous glutamatergic currents and GABAergic currents of GHRH neurons [27] were recorded as shown in Fig. 8A and 8C. Glutamatergic (recorded at −70 mV, Fig. 8A) and GABAergic (recorded at −30 mV, Fig. 8C) currents seemed unchanged by the superfusion with ghrelin (10 nM). It was found that ghrelin did not shift the cumulative distribution of the amplitudes and of the inter-event intervals of either the glutamatergic currents (n = 11, Fig. 8B), or the GABAergic currents (n = 6, Fig. 8D) in GHRH neurons. In these experiments, ghrelin did not modify the kinetics of the synaptic currents (data not shown). A synthetic GHS, JMV1843 100 nM, did not modify the spontaneous GABAergic and glutamatergic currents of GHRH-GFP neurons (n = 6, data not shown). Thus, fast synaptic transmission at GHRH neurons is unaffected by ghrelin.

### Ghrelin Had a Direct Depolarizing Effect on GHRH Neurons

**Figure 9:**
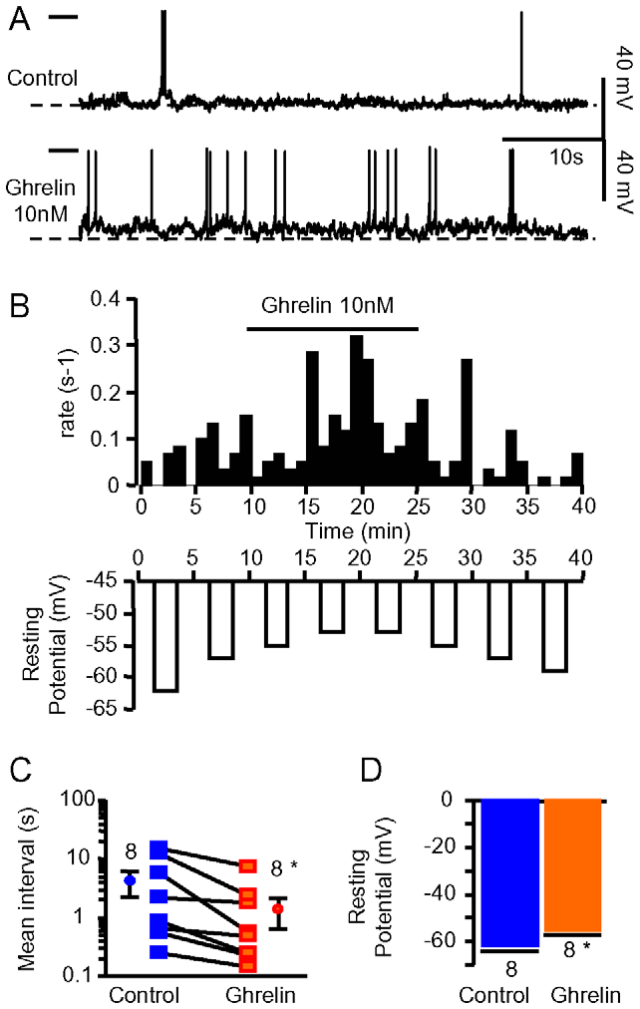
Ghrelin changed the excitability of GHRH neurons. ***A***, recordings from a GHRH neuron in the absence and presence of 10 nM ghrelin, in the perforated patch-clamp configuration. ***B***, time course of the effect of ghrelin 10 nM on the firing rate (upper graph) and on the resting potential (lower graph) of the GHRH neuron shown in ***A***. ***C***, summary of the effects of ghrelin 10 nM on the mean action potential intervals in GHRH neurons recorded in the perforated patch-clamp configuration. ***D***, mean amplitude of the resting potential in GHRH neurons in the absence and presence of 10 nM ghrelin (same experiments as in ***C***). Bars and lines are the means and the sem of the numbers of experiments indicated. Statistical difference (p<0.05, paired student-*t* test) with the control level is indicated.

The signature of the neuromodulatory effect of ghrelin on GHRH neurons was further investigated with the perforated patch-clamp technique [49], where amplitudes and kinetics of action potentials can be quantified (as shown by the individual traces of Fig. 9A). In the recording of Fig. 9A–B, the spontaneous action potentials of a GHRH neuron were collected under control conditions in the current-clamp mode (0 pA). Superfusion of the slice with ghrelin 10 nM increased the firing rate of the neuron (Fig. 9B, top panel) and this stimulation was mirrored by a decrease in the resting membrane potential (Fig. 9B, bottom panel). In similar experiments, ghrelin consistently decreased the mean action potentials intervals (from 4.31±2.0 s to 1.40±0.77 s, n = 8, p<0.05, paired student t-test: Fig. 9C), without changing the skewness of the interval distribution (data not shown), consistent with the results from extracellular recordings. Ghrelin consistently depolarized GHRH neurons (from −61.88±2.81 mV to −55.31±2.15 mV, n = 8, p<0.005, paired student t-test: Fig. 9D) and did not alter the parameters of the action potentials (Table 1). Similar results were found when ghrelin was applied in the presence of the AMPA/kainate antagonist DNQX (6,7-dinitroquinoxaline-2,3-dione, 15 µM) plus the GABA_A_ antagonist GABAzine 3 µM, which eliminated spontaneous synaptic depolarisations and hyperpolarizations (data not shown). These experiments showed that ghrelin modified an intrinsic ionic current of GHRH neurons. This was not studied further, however, because of space-clamp limitations [12].

**Table 1:** Effects of ghrelin on the properties of action potentials in GHRH neurons (n = 8).

	Parameters	Control	Ghrelin 10 nM
Amplitude (mV)	Threshold	−45.7±3.4	−43.9±2.2
	Peak	4.6±3.0	5.3±2.0
	After hyperpolarisation	−54.0±3.8	−51.6±3.1
	Peak - threshold	49.8±3.5	49.7±2.9
	AHP - threshold	−7.7±1.1	−8.8±1.0
Duration (ms)	Time-to-peak	5.92±0.46	6.17±0.34
	Time-to-AHP	12.20±0.94	12.69±0.88
	Half-width	1.60±0.13	1.63±0.19

## Discussion

The GH axis is a well-known target for GHS and there is evidence that GHS can stimulate GHRH secretion [1], [4], [15], [22]. Our direct recordings of identified GHRH neurons in GHRH-GFP mice have confirmed that ghrelin enhances their spontaneous firing rate, providing a direct explanation for the hypothalamic effect of GHS on the GH axis. This stimulation was direct, required GHSR, phospholipase C and voltage-dependent calcium channels, and paralleled other effects related to the modulation of NPY neuronal activity in the arcuate nucleus [2]. Thus, the growth axis and the appetite network have independent hypothalamic sensors for ghrelin, despite the fact that they overlap within the arcuate nucleus.

Ghrelin exerted a direct stimulation on GHRH neurons and, importantly, did not modify spontaneous synaptic currents. This is unlike the muscarinic M1-mediated modulations of GHRH neurons [27], and consistent with the observation that a muscarinic antagonist, atropine, does not blunt the effect of ghrelin (unpublished data). The stimulatory effect of ghrelin was mimicked by GHSR agonists and fully antagonized by a GHSR antagonist [30]–[33]. It was interesting that the GHSR antagonist JMV3002 did not modify the spontaneous activity of GHRH neurons, suggesting that ghrelin responsiveness may normally require acute activation, and arguing against a constitutive activity of unliganded GHSR [50]. The effector of the GHSR in GHRH neurons was likely to be phospholipase C, since the stimulation of the firing rate induced by ghrelin was prevented by U-73122, a pharmacological blocker of the hydrolysis of phosphatidylinositol 4,5-bisphosphate to inositol phosphates [38]–[40]. Activation of the phospholipase C pathway generally enhances intracellular calcium dynamics, and indeed, ghrelin elicits calcium transients in isolated hypothalamic neurons from immature animals, including some GHRH-positive neurons [20], [21]. In the present study, the effect of ghrelin was antagonised by voltage-dependent calcium channels blockers (with either Ni^2+^ or Cd^2+^), but not by neurotransmission disruption (with the combination of CNQX + GABAzine). Therefore, it is likely that ghrelin required and/or targeted high and low voltage-activated calcium channels in GHRH neurons. In comparison, N-type channels were involved in the generation of the calcium transients by ghrelin in cultures of NPY neurons [51]. A requirement for calcium channels might not be ubiquitous because ghrelin enhanced the firing rate of unidentified neurons of the arcuate nucleus in calcium-depleted medium [12], [52], [53]. This treatment not only slows down neurotransmission, but eliminates voltage-dependent calcium influx as well. For a comparison, a calcium-deprived medium profoundly altered the action potentials kinetics in GHRH neurons, which became silent within minutes, precluding further studies (unpublished data). Perforated patch clamp results showed that ghrelin depolarized GHRH neurons in a tonic manner, and did not significantly modify the kinetics of the spontaneous action potentials. A stimulation of low voltage-activated calcium channels might account for this depolarization, although other mechanisms might be involved. Indeed, calcium influx controls a variety of background conductances, including some Gd^3+^-sensitive transient receptor potential channels [41], [44]. Furthermore, it was interesting to notice that narrow range blockers (of calcium channels) were as efficient in eliminating the ghrelin stimulation than the broader range compounds Gd^3+^ and flufenamic acid [40], [43], [44]. Future work will dissect out the molecular events linking membrane and internal targets of ghrelin, notably mitochondria, in GHRH neurons [42]. The important role of calcium ions might explain why ghrelin less consistently enhanced the firing rate of GHRH neurons of aged mice. Indeed, calcium buffering is impaired in aged neurons [54], and some of them might not tolerate the elevation of the firing rate (the present study) and the elevation in intracellular calcium [21] induced by ghrelin.

Ghrelin increased the spontaneous firing rate, but did not modify either the firing pattern or the synchronisation amongst GHRH neurons. This characterizes a simple mechanism for the hypothalamic stimulation of the GH axis. Electrical activation of the arcuate nucleus, which recruits GHRH neurons, is a relevant trigger of GH secretion [55], and *in vivo* GH secretion is potentiated with increasing duration, but not increasing frequency, of electrical-field stimulations of the arcuate nucleus [55]. The effect of ghrelin is similar, since it promotes a sustained electrical activity of GHRH neurons. Synchronisation of the GHRH network might be facultative for factors, like ghrelin, which enhance the amplitude and not the frequency of GH pulses [1]. Some mathematical models have incorporated an antagonistic effect of ghrelin on the hypothalamic effects of somatostatin [1], but this is not essential, since in the mouse, the stimulatory effect of ghrelin on GHRH neurons was observed in the absence of somatostatin.

There is debate as to the origin of ghrelin that exerts a hypothalamic effect. Peripheral ghrelin crosses the blood brain barrier [56], [57], as seen in the arcuate nucleus where GHRH cells bodies are located [28]. Peripheral ghrelin or GHS induce rapid *c-fos* expression in GHRH neurons [16], [17], [58]. Thus, ghrelin is clearly capable of acting as a hormone to activate the GH axis at the hypothalamic level. Peripheral ghrelin can also stimulate GH cells directly, and therefore promote a synergy of effects at the pituitary gland level [1], [4]. In addition, the ghrelin-containing synapses, found within the arcuate nucleus [12], might have a specific effect at the hypothalamic level of the GH axis. Their origin remains unclear, however, and they might represent a small, or a very specialized, population as there is very little measurable ghrelin in the hypothalamus. Moreover, whereas ghrelin-positive synapses connect NPY neurons and GABAergic synapses [12], it is unknown if ghrelin neurons synapse onto GHRH neurons. If so, their basal tonic activity would be expected to be low in acute brain slices since JMV3002, the GHSR antagonist [33], did not change the firing rate of GHRH neurons. Importantly, while GHRH might have some properties of a GHSR agonist [59]–[61], mouse GHRH did not mimic the stimulatory effect of ghrelin on GHRH neurons (unpublished data).

This direct modulation of GHRH neurons by ghrelin parallels the direct effect of ghrelin on NPY neurons, which orchestrates the activity of the appetite network [2]. GABA and NPY, two products of NPY neurons, were not involved in the effect of ghrelin on GHRH neurons (present study), and AgRP is thought to be ineffective on the GH axis, notably at the hypothalamic level [62]. In addition, GHRH neurons are unlikely to regulate NPY neurons directly because they project towards the median eminence, but not to the arcuate nucleus [22]. Although an attractive concept, our results do not support the notion of a co-ordination of functions by ghrelin at multiple targets in the arcuate nucleus. Instead, ghrelin can modulate independent targets and regulate body functions in an independent manner. These findings do not exclude the fact that other products might synchronize the activity of the GH axis and the feeding circuits [25], [26]. Our data demonstrating that Y2 receptor activation stimulated the electrical activity of identified GHRH neurons is quite provocative in this respect, since these same Y2 receptors are mandatory for an adaptation of GHRH neurons to prolonged fasting [25], [26]. We speculate that the growth axis and the appetite network do overlap under some circumstances, perhaps following afferent rewiring [1]. Alternately, the different hypothalamic effects of ghrelin might evolve independently during development, since ghrelin and GHSR are expressed at early stages in life [34], [35], [63]. GHRH neurons were responsive to ghrelin in 6 day-old mice and this mechanism might participate in the GH secretion elicited upon ghrelin injection in immature rats, aged 1–3 weeks [64], [65].

Nitric oxide (NO), another product of NPY neurons [66], [67] might orchestrate the activity of the arcuate nucleus, without the need for synaptic rewiring. Indeed, a NO synthase inhibitor antagonizes the effect of ghrelin on food intake [68], and NO synthase mediate the effects of ghrelin in the pituitary gland [69]. However, ghrelin and NO have opposite effects on excitability in the arcuate nucleus (our unpublished data) [70], suggesting that NO release cannot account for the major molecular effects of ghrelin.

Ghrelin directly activated GHRH neurons, and this modulation obviously concerns the GH axis, and does not require NPY neurons involved in feeding. Although the same receptors, GHSR, are involved in both regulatory effects, there might be differences in the subsequent transduction pathways underlying the effects of ghrelin in GHRH neurons and NPY neurons. For a comparison, GHSR is expressed in both GH cells and GHRH neurons [1], [4], but the effects of ghrelin are not identical in both these cell types. Insights into these mechanisms could assist in the development of pharmacological agents in the treatment of feeding disorders or GH deficiencies [4]. Ghrelin receptors can be found at the NPY nerve terminals, accounting for presynaptic modulation of POMC- and CRH-neurons [12]. It is not known if GHSR also localize to GHRH nerve terminals. Future studies will be needed to address the mechanisms of action of ghrelin at the median eminence, characterized by its abundance of fenestrated blood vessels. At this location, ghrelin might modulate the activity of nerve terminals relevant to the GH axis.

## Materials and Methods

All animal studies complied with the animal welfare guidelines of the European Community, and/or UK Home Office guidelines, as appropriate. They were approved by the Direction of Veterinary departments of Herault, France (agreement number 34.251) and the Languedoc Roussillon Institutional Animal Care and Use Committee (#CE-LR-0818).

### Slice Preparation for Electrophysiological Recordings

Adult 12–16 week-old, GHRH-GFP mice [27], [28] were anesthetized by isoflurane inhalation, killed by decapitation, and brains quickly removed into cold (0–2°C) solution-1 [in mM; 92 NMDG-Cl, 2.3 KCl, 1 CaCl_2_, 6 MgCl_2_, 26 NaHCO_3_, 1.2 KH_2_PO_4_, 25 glucose, 0.2 ascorbic acid, 0.2 thiourea; pH 7.4 gassed with 95% CO_2_, 5% O_2_]. Sagittal sections (300 µm-thick) were cut with a microtome (Integraslice 7550, Campden Inst., UK) and stored at 34°C in solution-2 [in mM; 115 NaCl, 2.5 KCl, 1 CaCl_2_, 4 MgCl_2_, 26 NaHCO_3_, 1.25 NaH_2_PO_4_, 25 glucose, 0.2 ascorbic acid, 0.2 thiourea; pH 7.4, gassed with 95% CO2, 5% O_2_] for at least 45 min. When indicated, young, 6 days-old GHRH-GFP male mice (Gouty-Colomer et al. submitted), or aged, 22–30 months-old GHRH-GFP male mice [29] were investigated without modifications of the method.

### Patch-Clamp Recordings

Slices were immobilized with a nylon grid in a submersion chamber on the stage of an upright microscope (Axioskop FS2, Carl Zeiss) and superfused with solution-3 [in mM; 125 NaCl, 2.5 KCl, 2 CaCl_2_, 1 MgCl_2_, 26 NaHCO_3_, 1.25 NaH_2_PO_4_, 12 glucose; pH 7.4, gassed with 95% CO_2_, 5% O_2_] at a rate of ∼1.5 ml/min for at least 15 min at 30–32°C. A variant was used when NiCl_2_, CdCl_2_ and GdCl_3_ were included [in mM; 138 NaCl, 2.5 KCl, 2 CaCl_2_, 1 MgCl_2_, 3 NaHCO_3_, 1.25 NaH_2_PO_4_, 10 HEPES, 12 glucose; pH 7.4, adjusted with NaOH, saturated with 100% O_2_]. Slices were viewed with a x63 immersion objective and Nomarski differential interference contrast optics. Infrared differential interference contrast illumination was used to visualize neurons deeper in the slices and the images captured with an infrared camera (C2400, Hamamatsu Photonics, Massy, France). Borosilicate glass pipettes were connected to the head stage of an EPC-9/2 amplifier (HEKA, Lambrecht, Germany) to acquire and store data using Pulse 8.09 software. Agonists were bath-applied, and solutions were changed by switching the supply of the perfusion system from one to another. Typically, the effect of ghrelin 10 nM reached steady-state within 6–8 minutes, and the mean recovery time from this effect was ∼25 minutes. Activity was recorded for at least 4 min at steady state under each condition.

*For extracellular recordings* of spontaneous action potentials, pipettes (5–7 MΩ) were filled with (in mM), 130 NaCl, 2.5 KCl, 10 HEPES, 10 Glucose, 2 CaCl_2_, 1 MgCl_2_, pH 7.4 with NaOH (295 mOsm adjusted with NaCl). Neuronal activity was recorded in the voltage clamp mode (0 mV) of the loose-patch configuration [27]. *For whole cell recordings*, pipettes (6–8 MΩ) were filled with (in mM), 2.25 KCl, 125.3 KMeSO_3_, 10 HEPES, 0.1 EGTA acid, 1 MgCl_2_, 2 MgATP, 0.5 Na-GTP, 5 Na_2_-phospocreatine, 2 Na-pyruvate, 2 malate, pH 7.2 with KOH (295 mOsm adjusted with KMeSO_3_). Voltage- or current-clamp recordings were then performed as described [27]. *For perforated patch-clamp recordings*, gramicidin-D (50 mg/ml in dimethylsulfoxide) was dissolved at 50 µg/ml in the internal medium. The tips of the recording electrodes (4–6 MΩ) were filled with the protein-free solution, and backfilled with the antibiotic-containing medium [49]. Perforation of the membrane patch was evaluated in the cell-attached configuration under current-clamp at 0 pA, and recordings were started when resting membrane potential was <−50 mV and action potential amplitude was >50 mV.

### Chemicals

Chemicals were from Sigma-Aldrich (L'isle d'Abeau, France) except d-Glucose (Euromedex, France); tetrodotoxin (Latoxan, France); BIIE 0246 (Tocris bioscience, Bristol, UK). U-73122 and U-73343 were prepared as 10 mM stock solutions in DMSO and kept frozen at −20°C until use; flufenamic acid was prepared daily as 0.5 M stock solutions in DMSO.

### Data Analysis

Standard off-line detection of spontaneous events (action potentials or synaptic currents) were performed with Axograph 4.0 (Axon Instruments Inc., Foster City, CA). In brief, a template was generated and used to scan the raw trace for similar waveforms. All matching events were stored and, when present, false positive events were discarded, either manually or automatically on the basis of their amplitude or kinetics. Other calculations and analysis were performed with IgorPro (Wavemetrics, Lake Oswego, OR). The cumulative distributions were generated from stretches of >4 minutes-long series of data (such as amplitude or frequency of either action potentials or synaptic currents) recorded at steady state. The distribution histogram of this stretch was calculated using the appropriate binning interval (common to all the experiments) and normalized to the number of events. Cumulated distributions of the normalized data were then generated using the same binning intervals. This presentation allowed the statistical analysis (using the Kolmogorov-Smirnoff test, see below) and permitted inspection of the distributions. The modulation of GHRH neurons essentially shifted the position of the cumulated distributions in either direction, and did not modify the mean slope of the distributions. Accordingly, the frequency at the half maximum of the cumulated distributions was used as an index of the position of the cumulated distribution.

Auto-correlograms were generated as follows: we constructed a counting variable *N(t,ds)* corresponding to the number of events falling at distance *t* from an other event of the signal, within bin *ds* [71]. The histogram of this counting variable, once suitably normalised for bin size *ds* and total measurement time *T*, constitutes the auto-correlogram. To compute the corresponding confidence limits, we relied on Brillinger results [72], according to whom the square root of the cross-correlation distribution can be approximated to a normal distribution of mean *P_0_*, the mean density of the process, and of sem *1/(4 ds T)^1/2^*. A 95% confidence interval was thus computed as *P_0_±1.96/(4 ds T)^1/2^*. Note that boundary effects inherent for finite data were corrected for, by sub-weighting extreme values appropriately. Crosscorrelograms were computed in a similar way. The approximate distribution used for confidence intervals being now, mean (*P_0_P_1_)^1/2^*, with *P_0_* and *P_1_* the mean density of the two processes, and of sem *1/(4 ds T)^1/2^*. A 95% confidence interval was also computed as (*P_0_P_1_)^1/2^±1.96/(4 ds T)^1/2^*. The temporal organisation of stretches of action potentials was also evaluated with a statistical test, which required a randomisation of the neuronal activity, based on the statistics of the activity itself. The procedure was to use the inter-event intervals of the spontaneous action potentials and draw, from this empirical distribution, a shuffled sequence of random intervals. Thus, this artificial signal was totally decorrelated and had the same histogram signature than the empirical series of data. Comparisons between cross-correlograms generated with the artificial and the empirical data were then performed.

### Statistics

In each experiment, the Kolmogorov-Smirnoff (KS) test was used to test the statistical difference between two distributions obtained at steady-state (typically in the absence and in the presence of an agonist). Data were then expressed as mean ± standard-error-of-the-mean (sem) and the averaged distributions were compared at each abscissa value with a paired student*-t* test, to delineate the ranges of differences between untreated and treated distributions. p<0.05 was taken as significant (*ns*, not significant). Mean distributions are represented as lines connecting the mean values (symbols) and error bars represent the sem. For clarity, only part of the mean ± sem values are shown in the graphs.

## Supporting Information

Figure S1 Ghrelin changed the firing rate but not the firing pattern of GHRH neurons. A, on average, ghrelin (10 nM) strongly diminished the mean intervals of action currents in GHRH neurons from adult males, and had no significant effect on the skewness of the density histograms of these intervals, suggesting that it did not shift the range of firing rates of GHRH neurons. B–C, auto-correlogram analysis of the action currents intervals in the absence and presence of ghrelin 10 nM. B, analysis of a typical individual experiment where the autocorrelograms of the action current interval distributions are shown. Superimposed are the 95%-confidence boundaries of random distributions computed from the data sets. The firing rate of the GHRH neuron was enhanced by ghrelin (as evidenced by the upward shift of the distribution), without a change in the bursting behaviour (similar monotonous distributions), and the distributions were framed within the boundaries of random distributions. C, mean autocorrelogram distributions where solid lines are the means of 24 experiments. Statistical significance (paired student-t test) between curves was found in a very narrow range of action current intervals (−0.3 to +0.3 s, shaded grey area), in accordance with the conclusion that ghrelin increased firing rates without changing its firing patterns. These findings agree with previous observations that GH secretion evoqued by electric stimulation of the arcuate nucleus is potentiated with increasing burst durations, but not with increasing stimuli frequency [43].
(0.14 MB TIF)

Figure S2 The stimulatory effect of ghrelin on GHRH neurons changed during development. A, time course of an experiment where a single GHRH neuron was recorded from an immature GHRH male mouse (PN6). C, simultaneous recordings of GHRH neurons from an aged (24 months-old, C) male GHRH-GFP mouse, and where 10 nM ghrelin enhanced the activity of one neuron, but induced a transient inhibitory effect in the other GHRH neuron. B&D, summaries of the effects of ghrelin (10 nM) on the distributions of action current frequencies in GHRH neurons from immature PN6 (B) and aged 22–30 months-old (D) male GHRH-GFP mice. Note that the effects of ghrelin on GHRH neurons were heterogeneous in aged animals. Symbols and lines are the means and the sem of the numbers of experiments indicated. Statistical significances (paired student-t test) between curves are shown by the grey areas.
(0.25 MB TIF)
