# Supplementary figures and images for: Ghrelin Stimulation of Growth Hormone-Releasing Hormone Neurons Is Direct in the Arcuate Nucleus
Source: PLoS One. 2010 Feb 11;5(2):e9159. doi: 10.1371/journal.pone.0009159 (PMC2820089; doi:10.1371/journal.pone.0009159)

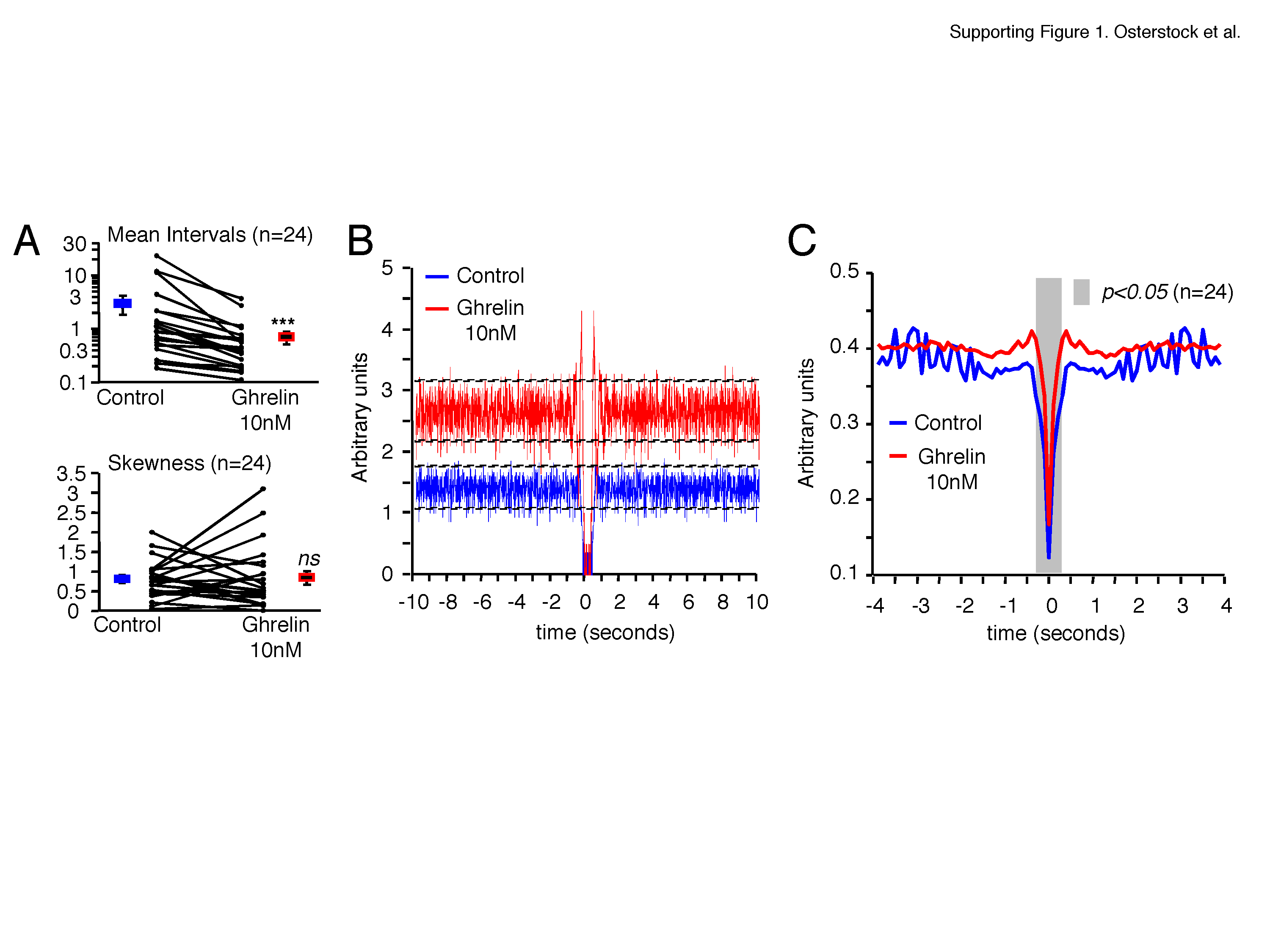

Supplement: Figure S1 — Ghrelin changed the firing rate but not the firing pattern of GHRH neurons. A, on average, ghrelin (10 nM) strongly diminished the mean intervals of action currents in GHRH neurons from adult males, and had no significant effect on the skewness of the density histograms of these intervals, suggesting that it did not shift the range of firing rates of GHRH neurons. B–C, auto-correlogram analysis of the action currents intervals in the absence and presence of ghrelin 10 nM. B, analysis of a typical individual experiment where the autocorrelograms of the action current interval distributions are shown. Superimposed are the 95%-confidence boundaries of random distributions computed from the data sets. The firing rate of the GHRH neuron was enhanced by ghrelin (as evidenced by the upward shift of the distribution), without a change in the bursting behaviour (similar monotonous distributions), and the distributions were framed within the boundaries of random distributions. C, mean autocorrelogram distributions where solid lines are the means of 24 experiments. Statistical significance (paired student-t test) between curves was found in a very narrow range of action current intervals (−0.3 to +0.3 s, shaded grey area), in accordance with the conclusion that ghrelin increased firing rates without changing its firing patterns. These findings agree with previous observations that GH secretion evoqued by electric stimulation of the arcuate nucleus is potentiated with increasing burst durations, but not with increasing stimuli frequency [43]. (0.14 MB TIF) [file pone.0009159.s001.tif]

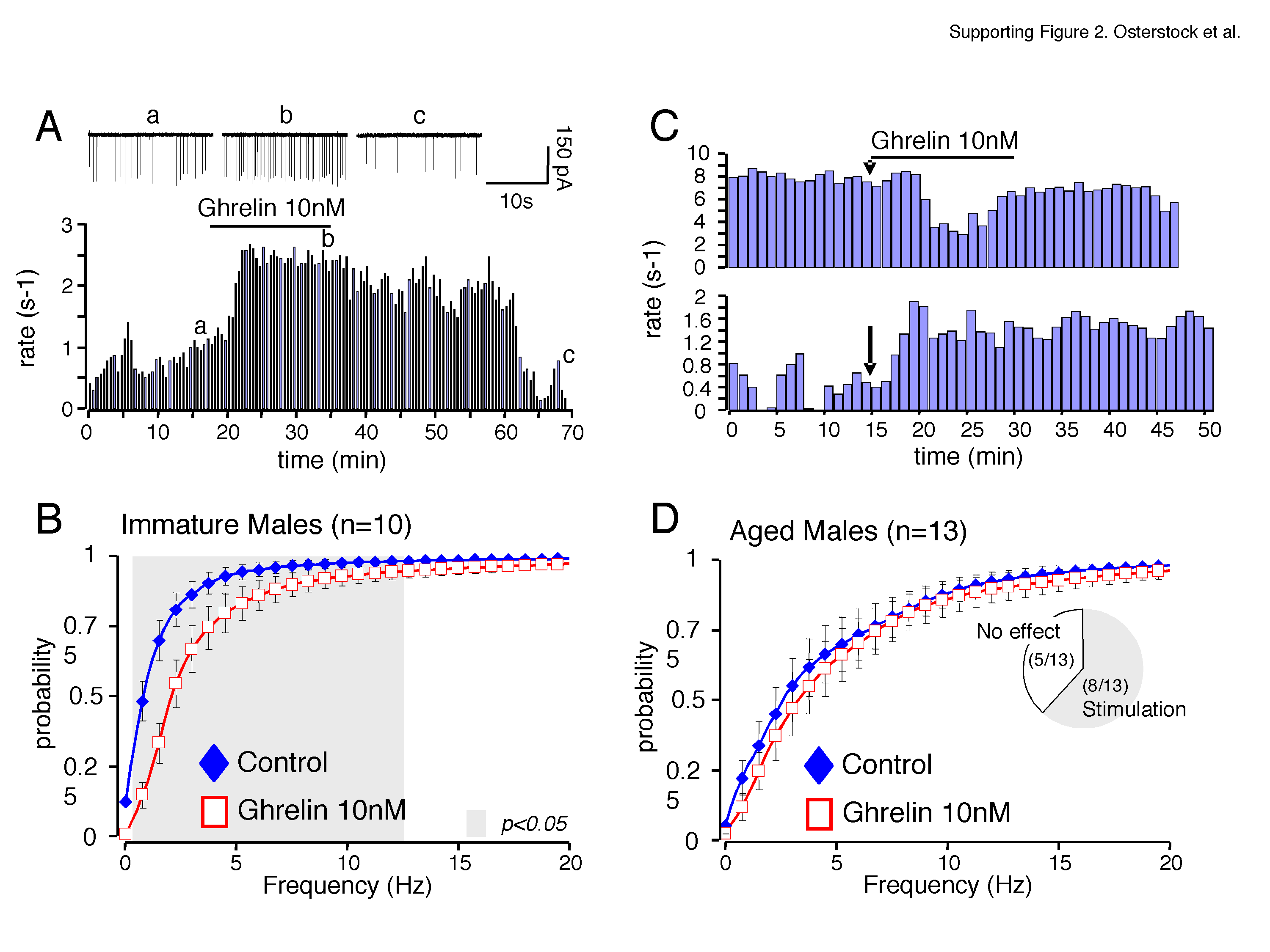

Supplement: Figure S2 — The stimulatory effect of ghrelin on GHRH neurons changed during development. A, time course of an experiment where a single GHRH neuron was recorded from an immature GHRH male mouse (PN6). C, simultaneous recordings of GHRH neurons from an aged (24 months-old, C) male GHRH-GFP mouse, and where 10 nM ghrelin enhanced the activity of one neuron, but induced a transient inhibitory effect in the other GHRH neuron. B&D, summaries of the effects of ghrelin (10 nM) on the distributions of action current frequencies in GHRH neurons from immature PN6 (B) and aged 22–30 months-old (D) male GHRH-GFP mice. Note that the effects of ghrelin on GHRH neurons were heterogeneous in aged animals. Symbols and lines are the means and the sem of the numbers of experiments indicated. Statistical significances (paired student-t test) between curves are shown by the grey areas. (0.25 MB TIF) [file pone.0009159.s002.tif]
